# Development and Application of a Cultivar-Specific Sequence-Characterized Amplified Region (SCAR) Marker for the Detection of *Chrysanthemum morifolium* Ramat. ‘Daboju’

**DOI:** 10.3390/plants11050604

**Published:** 2022-02-24

**Authors:** Yuchen Cai, Yadi Gao, Zhenhao Zhang, Huijie Liu, Yifan Wang, Yuxin Ma, Yixin Li, Shangguo Feng, Huizhong Wang

**Affiliations:** 1 College of Life and Environmental Science, Hangzhou Normal University, Hangzhou 310036, China; 2019111010003@stu.hznu.edu.cn (Y.C.); 2020111010014@stu.hznu.edu.cn (Y.G.); 2021111010013@stu.hznu.edu.cn (Z.Z.); liuhuijie@stu.hznu.edu.cn (H.L.); wangyifan@stu.hznu.edu.cn (Y.W.); myx@stu.hznu.edu.cn (Y.M.); liyixin@stu.hznu.edu.cn (Y.L.); 2 Zhejiang Provincial Key Laboratory for Genetic Improvement and Quality Control of Medicinal Plants, Hangzhou Normal University, Hangzhou 310036, China

**Keywords:** *Chrysanthemum morifolium*, cultivar identification, start codon-targeted polymorphism, sequence-characterized amplified region, marker development

## Abstract

*Chrysanthemummorifolium* Ramat. ‘Daboju’ is a *C. morifolium* cultivar with important ornamental and medicinal values, and is often used in the treatment of colds, blurred vision, dizziness, and itchy skin. As the morphological characteristics of *C. morifolium* ‘Daboju’ are very similar to those of other *C. morifolium* cultivars, they are often confused in practice. However, the medicinal value and practical use of *C. morifolium* depends on using the correct rapid and accurate identification of *C. morifolium* ‘Daboju’ and its differentiation from other, morphologically similar C. × morifolium cultivars. Twenty-one polymorphic start codon-targeted (SCoT) primers were amplified in 21 distinct *C. morifolium* cultivars. One cultivar-specific DNA marker was developed with the aim of the rapid and accurate identification of *C. morifolium* ‘Daboju’ and its differentiation from other, similar *C. morifolium* cultivars. Twenty-one polymorphic start codon-targeted (SCoT) primers were amplified in 21 distinct *C. morifolium* cultivars. One cultivar-specific 385-bp amplicon (named SCoT36-385), amplified only in *C. morifolium* ‘Daboju’ (and in all samples of this cultivar), was identified, cloned, and sequenced. Subsequently, a sequence-characterized amplified region (SCAR) marker (named DBJF/DBJR), generating a 360-bp amplicon, was developed from SCoT36-385 and tested for amplification in all 21 *C. morifolium* cultivars, ten *C. morifolium* ‘Daboju’ populations, and different simulated adulterations of ‘Daboju’ with other cultivars. The primers amplified the specific 360-bp-long DNA fragment in all the tested *C. morifolium* ‘Daboju’ samples but failed in the absence of ‘Daboju’. The detection limit of the SCAR primer pair (DBJF/DBJR) was 100 pg of DNA extracted from *C. morifolium* ‘Daboju’. Hence, this SCAR marker has a very high detection sensitivity, and can be used for accurate and rapid identification of *C. morifolium* ‘Daboju’. It can play an important role in ensuring the quality of medicinal preparations and protecting *C. morifolium* ‘Daboju’ germplasm resources in breeding programs and in identifying lines generated from this cultivar.

## 1. Introduction

Chrysanthemum, *Chrysanthemum morifolium* Ramat., belongs to the family Asteraceae, and it has a long history of cultivation and use as a popular ornamental and herbal medicine use and cultivation in China, South Korea, Thailand, and Japan [1,2]. As a traditional medicinal plant, *C. morifolium* flowers have significant pharmacological effects for treating headaches, dizziness, sore carbuncles, swelling, poison, and cold or wind heat [3]. As growing conditions and processing methods vary between production areas, different regions produce different medicinal preparations. *C. morifolium* cultivars classified as ‘Boju,’ ‘Chuju,’ ‘Gongju,’ ‘Hangju,’ or ‘Huaiju’ types, based on origin and processing methods, are legal Chinese medicinal materials, according to the 2015 edition of the Pharmacopoeia of the People’s Republic of China [3]. 

*C. morifolium* preparations are slightly cold in effect, with a sweet and bitter taste, with effects on wind, clearing heat, liver problems (ping mingmu tea), and for detoxification issues. Many studies have shown that the main active ingredients of chrysanthemum medicinal preparations include flavonoids, phenylpropanoids, and volatile oils, which have antiviral, anti-tumor, and anti-inflammatory effects [4,5,6]. Famous Chinese herbal medicine books, such as the ’Chinese Medicine Dictionary’ and ’Chinese Materia Medica,’ recorded that the ‘Boju’ type was the highest quality of medicinal chrysanthemum in China, and was mainly produced in Bozhou City, An’hui Province. In 2014, the Ministry of Agriculture of the People’s Republic of China officially approved the registration and awarded the protection of agricultural products from ‘Boju’ under geographical indication [3].

However, despite the wide variety of chrysanthemum cultivars, the morphological characteristics of cultivars used in Traditional Chinese Medicine (TCM) are relatively similar; with the mutual introduction and cultivation of the same cultivars in different regions, the classification of chrysanthemum cultivars has become quite confused at present, and the problem of ’synonyms’ and ’homonyms’ is very serious [2,7]. Studies have shown that different accessions of medicinal chrysanthemum exhibited great differences in terms of biochemical quality, yield, and other aspects, and that their medicinal value was also very different [8,9,10]. For example, cultivars of the ‘Boju’ type are often used as a classical herbal tea to treat colds, blurred vision, dizziness, and itchy skin in TCM [11,12]. 

In recent years, *C. morifolium* ‘Daboju,’ as an important cultivar of the ‘Boju’ type, has attracted extensive interest due to its unique phenolic and volatile components, and its anti-inflammatory and anti-allergenic biological activities [3,11]. However, driven by economic interests, some unscrupulous merchants have used chrysanthemum varieties of poorer quality when marketing preparations of ‘Daboju,’ leading to chaos in the ‘Daboju’ market and inconsistent quality of chrysanthemum products, seriously affecting the healthy development of the ‘Daboju’ industry [13]. Therefore, to better protect and utilize ‘Daboju’ resources, it is of great importance that a rapid and accurate method for the identification and authentication of ‘Daboju’ be established, to distinguish it from material from other cultivars being passed off as ‘Daboju’.

Initially, morphological [14,15], cellular [16], and biochemical markers [12,17] were often used as the major detection methods to distinguish different chrysanthemum cultivars. All these methods are difficult to operate, and are susceptible to environmental and developmental factors, and to plant sample adulteration. For example, some systems classify chrysanthemums according to their flower color, shape, or flowering period, but the use of all of these are restricted to the flowering stage of plant development. In contrast, the use of DNA molecular markers is an efficient and low-cost method for plant identification, and one that is independent of environmental and physiological conditions [18,19]. To date, many DNA molecular markers, including randomly amplified polymorphic DNA (RAPD), amplified fragment length polymorphism (AFLP), inter-simple sequence repeat (ISSR), sequence-related amplified polymorphism (SRAP), and simple sequence repeat (SSR), have been used widely used in genetic diversity assessment [20,21,22], phylogenetic analysis [23], genetic linkage map construction [24], QTL mapping [25,26], and phenotypic trait association analysis [27] of chrysanthemum germplasm resources. However, a number of studies have shown that the direct application of these markers to the molecular identification of plants is not ideal [28,29,30]. Sequence-characterized amplified region (SCAR), a class of reliable PCR-based DNA molecular marker, has been developed from a specific nucleotide sequence generated by certain traditional molecular markers, such as RAPDs, ISSRs, and AFLPs [31,32,33]. SCAR markers have been widely used in the molecular identification of plant species, cultivars, or traditional medicinal preparations due to their high stability and specificity, simple operation, and low cost [28,30,34,35,36,37].

In the present study, we developed a useful SCAR marker for ‘Daboju’ identification via the use of start codon-targeted (SCoT) primers, which are PCR-based gene-targeted markers, in which the SCoT primers are based on conserved regions flanking the translation start codon of genes [38]. At the same time, we established a sensitive and simple PCR-based method for the rapid molecular identification of ‘Daboju’ and its differentiation from other, closely related chrysanthemum cultivars.

## 2. Results

### 2.1. SCoT Analysis and Specific Locus Identification

**Figure 1:**
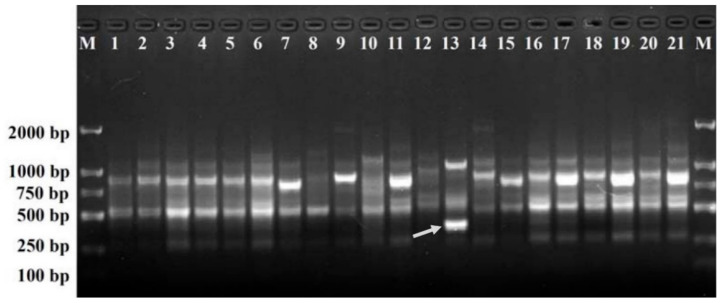
SCoT36 profiles of the 21 different *C. morifolium* cultivars (lanes 1–21). Lane M: Trans2K DNA markers with specified amplified product lengths (bp). Arrowheads represent specific amplified band in the *C. morifolium* ‘Daboju’ sample.

After the initial primer screening, a total of 21 SCoT primers, each generating clear and repeatable polymorphic patterns, were selected for further study (Table 1). The 21 SCoT primers generated 187 reliable loci with a range of 5−13 loci amplified by each primer, with an average of 8.90 loci per primer. Of these, 163 loci (87.17%) were polymorphic, ranging from 4 to 12 loci amplified per primer, with an average of 7.76 loci per primer. The percentage of polymorphic bands generated by each primer varied from 66.7% to 100.0%, with an average of 85.70%. Among the 163 polymorphic loci, only one amplicon (385-bp-long), amplified by primer SCoT36, was found to be present in the ‘Daboju’ sample but was absent from samples of all the other cultivars (Figure 1), which could therefore potentially be used for rapid and accurate molecular identification of ‘Daboju’ and the development of a specific SCAR marker specific for ‘Daboju’.

### 2.2. Sequence Analysis and SCAR Marker Development

**Figure 2:**
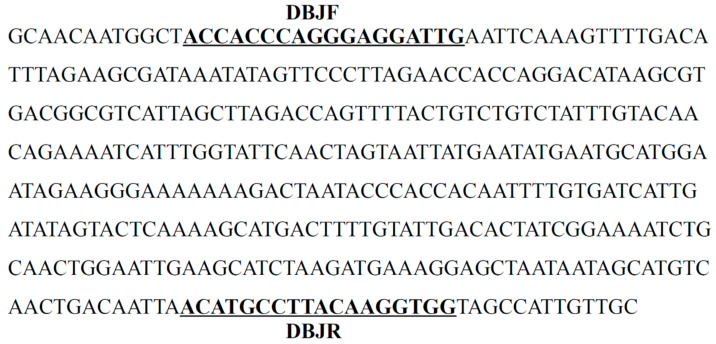
Nucleotide sequence of the SCoT marker specific to *C. morifolium* ‘Daboju’. The sequence was named SCoT36-360 and has been deposited in GenBank (Accession number: OL456174). The underlined bold sequences represent the forward primer (DBJF) and the reverse primer (DBJR).

The 385-bp amplicon specific to ‘Daboju,’ named SCoT36-385, was cloned, sequenced, and deposited in GenBank (GenBank accession number: OL456174). The specific nucleotide sequence of SCoT36-385 consisted of 63.12% A + T and 36.88% G + C, as shown in Figure 2. The BLASTN of the SCoT36-385 sequence did not show a similarity with other sequences in the GenBank database, and no repeats were detected. This ‘Daboju’-discriminating SCoT band was subsequently converted into a SCAR marker (primer pair named DBJF/DBJR) (Figure 2 and Table 2). The lengths of the forward and reverse primers for the SCAR marker DBJF/DBJR were 18- and 18-mer, respectively.

### 2.3. Amplification of the Designed SCAR Primers Designed

**Figure 3:**
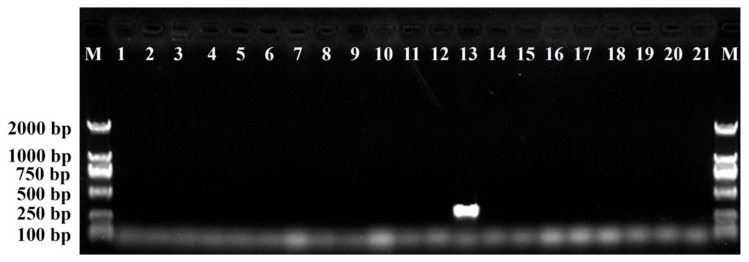
Amplification of the SCAR marker DBJF/DBJR primer pair developed in the genomic DNA of the 21 different *C. morifolium* cultivars (lanes 1–21). Lane M: Trans2K DNA marker with specified amplification product lengths (bp). A specific amplified band of 360-bp length was detected in the *C. morifolium* ‘Daboju’ sample.

**Figure 4:**
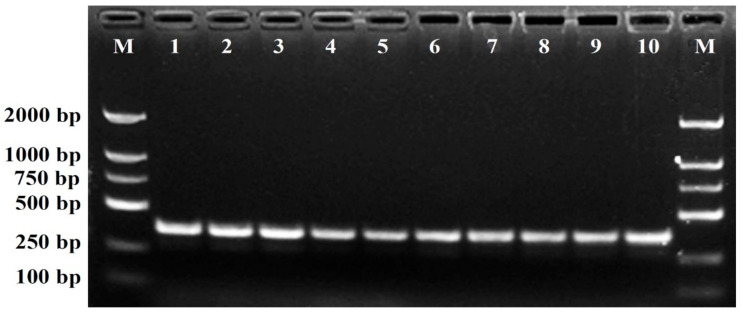
Amplification profiles of the primer pair DBJF/DBJR in the ten *C. morifolium* ‘Daboju’ samples (lanes 1–10). Lane M: Trans2K DNA marker with specified amplification product lengths (bp). The specific amplified bands of 360-bp length were detected in all the *C. morifolium* ‘Daboju’ individuals.

The designed SCAR primer pair DBJF/DBJR was then used to amplify genomic DNA from the 21 chrysanthemum cultivars at the optimum annealing temperature of 62 °C to evaluate the specificity of amplification. The amplification profile of the primer pair DBJF/DBJR is shown in Figure 3. The results showed that a clear, specific amplicon of 360 bp was generated from the ‘Daboju’ DNA samples by the primer pair, but no amplicons were detected from DNA samples from any of the other 20 chrysanthemum cultivars. The SCAR primer pair DBJF/DBJR did not contain the sequence of the original SCoT primer, thus giving rise to amplification products shorter than the original selected sequence, which should increase the specificity of amplification. Furthermore, the stability and specificity of the SCAR primer pair DBJF/DBJR were further validated in different DNA samples from ten ‘Daboju’ populations. The results showed the specific amplicon of the primer pair DBJF/DBJR at 360 bp with all ten samples confirming the identity of all ten ‘Daboju’ populations (Figure 4).

### 2.4. Sensitivity and Application of the Specific SCAR Marker

**Figure 5:**
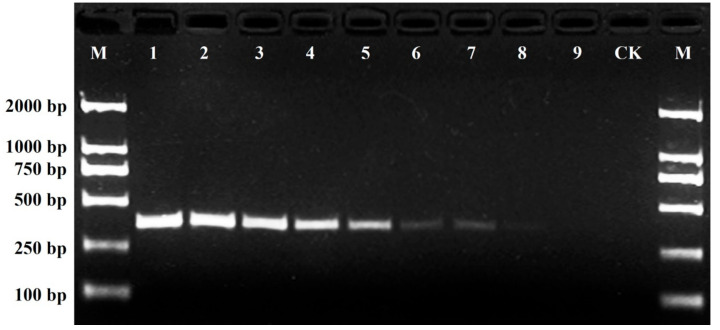
The sensitivity of the SCAR markers (DBJF/DBJR) with different *C. morifolium* ‘Daboju’ genomic DNA concentrations in a 20 µL PCR mixture. Lane M: Trans2K DNA Marker with specified amplification product band lengths (bp); lanes 1–9: 50 ng/µL, 40 ng/µL, 30 ng/µL, 20 ng/µL, 10 ng/µL, 5 ng/µL, 1 ng/µL, 100 pg/µL, and 10 pg/µL, respectively; lane CK: ddH_2_O, instead of the DNA template of *C. morifolium* ‘Daboju’.

**Figure 6:**
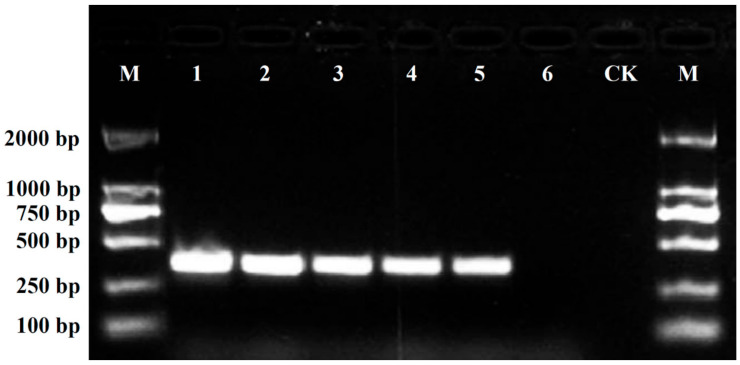
Amplification profiles of the primer pair DBJF/DBJR in genomic DNA composed of different combinations of *C. morifolium* cultivars. Lane M: Trans2K DNA markers with specified amplification product band lengths (bp); lanes 1–6: different proportions of adulteration of *C. morifolium* cultivars, with details of the adulterations provided in Table 3; lane CK: ddH_2_O, instead of the DNA template of *C. morifolium* cultivars.

The sensitivity of the SCAR marker DBJF/DBJR was evaluated using a dilution series of ‘Daboju’ genomic DNA samples. The results suggested that the sensitivity limit of the primers DBJF/DBJR was 100 pg of genomic DNA in the 20 μL PCR reaction mixture (Figure 5). Furthermore, the different simulated adulterations of ‘Daboju’ with tissue from other cultivars showed that only the simulated adulterated samples containing ‘Daboju’ tissue generated the 360-bp amplicon using the primers DBJF/DBJR, whereas this amplicon was not found in the adulterated samples of ‘Xiaoyangju,’ ‘Zaoxiaoyangju,’ ‘Dayangju,’ ‘Machengju,’ and ‘Hongxinju’ without ‘Daboju’ (Figure 6). Therefore, the SCAR marker DBJF/DBJR has been demonstrated to represent an effective tool with simple operation, high accuracy and sensitivity, and low economic cost to identify whether ‘Daboju’ was present in Chinese herbal medicinal materials for clinical applications.

## 3. Discussion

Accurate identification of plant cultivars is an important part of plant product quality control, especially in situations where suppliers may be intentionally providing material from the wrong cultivars [39]. ‘Daboju,’ one of the chrysanthemum cultivars with ornamental and medicinal use, occupies a prominent position in horticulture and TCM thanks to the important ornamental and medicinal values of its flowers. Substitution or adulteration of ‘Daboju’ preparations might damage the effectiveness of TCM treatments and even endanger the safety of consumers. Nevertheless, ‘Daboju’ and other medicinal chrysanthemum cultivars are very similar in terms of flower appearance and shape. As ’chrysanthemum’ is an umbrella term for many different chrysanthemum cultivars, which are very similar in appearance and closely related, accidental or deliberate adulteration or mislabeling is highly possible. Although different chrysanthemum cultivars can be distinguished by very subtle differences in flower morphology or color, chrysanthemums only bloom once a year under most climatic conditions. Hence, it is difficult to confirm the identity of these chrysanthemum cultivars quickly and accurately by traditional morphometric methods. Therefore, identification using qualitative molecular markers will contribute to our ability to identify chrysanthemum plants at any stage of development.

Up to now, many types of molecular markers, including RAPD, AFLP, SSR, ISSR, SRAP, and SCoT, have been used for genetic diversity evaluation, genetic relationship analysis, genetic map construction, and trait correlation analysis of chrysanthemum germplasm resources [10,22,24,26,40,41]. Although these DNA molecular markers have a wide range of applications for the DNA analysis of plants, their lack of reproducibility makes them unreliable for direct, rapid, and unequivocal identification of plant cultivars. 

To overcome these shortcomings, the SCAR marker, a more specific type of marker, was developed. SCAR markers have been widely used in the identification of cultivars, species, substitutes, and adulterants because they are specific to single loci and their PCR amplification is relatively insensitive to reaction conditions. Traditional DNA molecular markers, such as RAPD, ISSR, AFLP, SRAP, and SSR, can be transformed into SCAR markers based on sequence data, a process that improves the reproducibility of the PCR products of SCAR markers. In the present study, a specific SCAR marker was developed for the authentication of ‘Daboju,’ based on SCoT analysis. In recent years, SCAR markers have increasingly been used for the authentication of medicinal plants, such as *Ocimum tenuiflorum* [33], *Trapa natans* [42], *Lycium chinense* [43], and *Dendrobium officinale* [36]. In addition, SCAR markers have also been used to identify adulterants in commercially important plant-derived food and medicinal products, such as *Crocus sativus* [44], *Aconitum heterophyllum* and *Cyperus rotundus* [37], *Punica granatum* [30], and *Panax ginseng* [45].

As a molecular marker technique, generating high levels of polymorphism and capable of high stability, the recent introduction of SCoT markers has been widely welcomed in recent years [29]. Some SCAR markers, converted from SCoT primers, have been used for the identification of a number of plants [29,39,46,47]. In the present study, a specific 385-bp DNA sequence (SCoT36-385) was amplified by SCoT36, which could be used to identify ‘Daboju’ samples after being converted into a SCAR marker. In order to increase the stability and specificity of the amplification product, the sequence size of the PCR product amplified by SCAR primers was made slightly shorter than that of the original specific DNA fragment [29,30]. In this study, the specific band size obtained by the SCAR primers DBJF/DBJR was 360 bp, which was 25-bp shorter than the original specific SCoT sequence, SCoT36-385 (385 bp in length).

There are many cultivars of medicinal chrysanthemum, and the specific uses and medicinal values of different cultivars also vary. In order to ensure the clinical safety and effectiveness of preparations of chrysanthemum ‘Daboju,’ genuine raw material of ‘Daboju’ must be used. Therefore, it is necessary to establish a rapid, inexpensive, efficient, and accurate method by which to identify chrysanthemum ‘Daboju’ and distinguish it from other cultivars or from adulterated preparations. In recent years, DNA barcoding has gained popularity in species identification [48,49,50,51]. Its advantage is that the target sequence comes from a known region of the genome, which helps in evolutionary studies. However, DNA barcoding also has its disadvantages. For example, DNA barcoding requires the sequencing of every individual, which is expensive and time-consuming. Furthermore, DNA barcoding has a low success rate in distinguishing between cultivars. In contrast, SCAR marker technology is cheaper and takes less time than barcoding, and its specific primers are relatively easy to design. More importantly, SCAR marker primers are less affected by the external environment, and they have a very high success rate in the identification of interspecific, intraspecific, and adulterated products [30,39]. The SCAR marker primer pair DBJF/DBJR developed here could amplify specific DNA fragments of a certain length in ‘Daboju’ samples, whereas no DNA bands were amplified in samples of other chrysanthemum cultivars in the absence of a threshold concentration (100 pg DNA/20 μL) of ‘Daboju’. This finding showed that the SCAR primer pair DBJF/DBJR could be used for the identification of ‘Daboju’ and its distinction from other, similar chrysanthemum cultivars. Our results showed that the SCAR primer pair DBJF/DBJR could detect 100 pg of the ‘Daboju’ DNA template in a 20 µL PCR reaction mixture, which indicated that the identification sensitivity of DBJF/DBJR was very high, with only a small amount of sample DNA being needed to achieve rapid and accurate identification of ‘Daboju’. The development of cultivar-specific SCAR markers is more difficult than that of species-specific SCAR markers due to the closer genetic relationship between cultivars. It can be said that the cultivar-specific SCAR markers are complex to develop, requiring a large amount of work in screening, sequencing, design, and verification. However, once the SCAR marker is developed successfully, it is of great application value and significance with respect to its practical application.

## 4. Materials and Methods

### 4.1. Plant Materials

**Figure 7:**
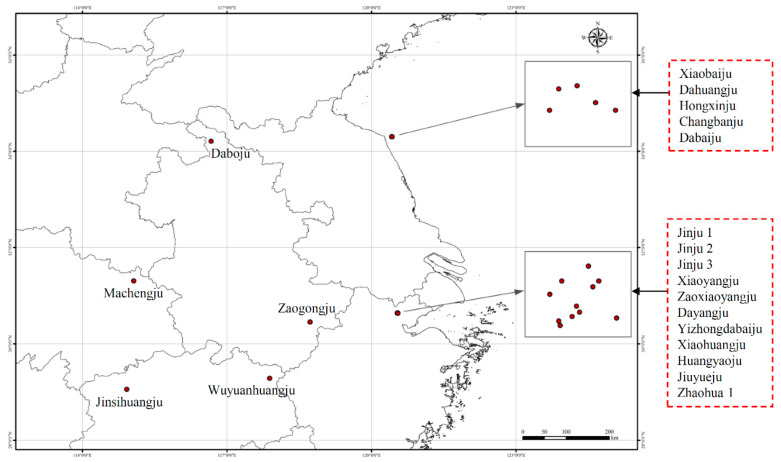
Sampling locations of 21 *C. morifolium* cultivars.

Twenty-one chrysanthemum cultivars, including ‘Jinju 1,’ ‘Jinju 2,’ ‘Jinju 3,’ ‘Xiaoyangju,’ ‘Zaoxiaoyangju,’ ‘Dayangju,’ ‘Yizhongdabaiju,’ ‘Xiaohuangju,’ ‘Huangyaoju,’ ‘Jiuyueju,’ ‘Zhaohua 1,’ ‘Jinsihuangju,’ ‘Daboju,’ ‘Zaogongju,’ ‘Wuyuanhuangju,’ ‘Xiaobaiju,’ ‘Machengju,’ ‘Dahuangju,’ ‘Hongxinju,‘Changbanju,’ and ‘Dabaiju,’ were collected from their main cultivation areas (Figure 7) for SCoT analysis by pooling fresh leaves from at least six individuals of each cultivar (Table 4). These plant materials were preserved and cultivated at the Tongxiang Chrysanthemum Experimental Field of the Zhejiang Provincial Key Laboratory for Genetic Improvement and Quality Control of Medicinal Plants, Hangzhou Normal University, Zhejiang Province, China. Voucher samples were deposited at the Zhejiang Provincial Key Laboratory for Genetic Improvement and Quality Control of Medicinal Plants. Relevant morphological features of flowers of samples are shown inFigure 8, respectively. In addition, tissue samples from 10 ‘Daboju’ populations (Table 5) collected from Zhejiang and An’hui provinces (Figure 9) were selected for validating the SCAR marker, using fresh leaves pooled from at least eight individuals of each population.

### 4.2. DNA Extraction

Total genomic DNA preparations were extracted after pooling fresh leaves from six or more different individuals of each cultivar, using the Plant Genomic DNA Extraction Kit (Shanghai Sangon Biological Engineering Technology and Service Co. Ltd., Shanghai, China). The integrity and quality of the DNA preparations were evaluated by electrophoresis on 1.0% (*w/v*) agarose gel, and the concentration of DNA was determined using a UV spectrophotometer.

### 4.3. PCR Amplification with SCoT Primers

Thirty-six PCR primers for SCoT analysis were selected from the study of Collard and MacKill [38]. SCoT-PCR was performed in 20 μL reaction volumes containing 10 μL of 2×*EasyTaq* PCR SuperMix (Beijing TransGen Biotech Co., Ltd., Beijing, China), 1 μL of genomic DNA template (50 ng), 1 μL of each primer (10 μM), and 8 μL of ddH_2_O. The PCR reaction was performed as follows: 94 °C for 5 min, 35 cycles of 94 °C for 1 min, 50–60 °C for 50 s (depending on the optimum annealing temperature of each primer), and 72 °C for 1.5 min, followed by extension at 72 °C for 10 min. The PCR products were analyzed by electrophoresis on 1.5% (*w/v*) agarose gels. The amplified DNA bands were stained with GelStain (Beijing TransGen Biotech Co., Ltd., Beijing, China) and the images photographed on a UV transilluminator. To check for reproducibility of results, PCR reactions were repeated at least three times.

### 4.4. Analysis of SCoT Profiles, and Selection of Specific SCoT Bands

In order to count the number of primer-amplified loci and to evaluate the degree of polymorphism of primer-amplified DNA bands, the number of amplified bands was calculated by Quantity One software (Version 4.6.2, Bio-Rad Technical Service Department, USA) with manual correction. The bands that were reproducible and unambiguous were marked as ‘1’ and the absence of a band at the same locus was scored as ‘0.’ Any DNA band present in a specific cultivar and absent in all the other cultivars at the same locus was defined as a cultivar-specific marker.

### 4.5. Cloning and Sequencing of Specific SCoT Fragments

A selected cultivar-specific band was extracted and purified from an agarose gel using a SanPrep Column DNA Gel Extraction Kit (Shanghai Sangon Biological Engineering Technology and Service Co. Ltd., Shanghai, China). The purified DNA fragment was cloned into the pMD^TM^19-T vector (Takara Co., Ltd., Beijing, China) according to the manufacturer’s protocol, transformed into DH5α *Escherichia coli competent* cells, and cultured at 37 °C overnight. The recombinant plasmids were selected by colony PCR, and clones with correct-sized inserts were sequenced bidirectionally, using M13 universal primers by Shanghai Sunny Biotechnology Co. Ltd. (Shanghai, China).

### 4.6. Sequence Data Analysis and SCAR Marker Development

The online tool VecScreen (https://www.ncbi.nlm.nih.gov/tools/vecscreen/, accessed on 28 December 2021) was used to remove vector sequences from the obtained sequence, and the nucleotide database of the BLASTN program (https://blast.ncbi.nlm.nih.gov/Blast.cgi, accessed on 28 December 2021) was used to identify the similarities between sequences. Ultimately, the sequence obtained was submitted to the GenBank database (GenBank accession number: OL456174) [52]. The SCAR primer pair (named DBJF/DBJR) was designed based on the specific 385-bp nucleotide sequence of the ‘Daboju’-specific SCoT36-385 amplicon, obtained using Primer Premier 5 software [53] (Table 2), and synthesized by Shanghai Sangon Biological Engineering Technology and Service Co. Ltd., Shanghai, China.

### 4.7. Specificity of the SCAR Marker

First, the specificity of the SCAR marker was evaluated by PCR amplification of the DNA of the 21 medicinal chrysanthemum cultivars (Table 4). Furthermore, 10 selected ‘Daboju’ populations were used to further validate the SCAR marker developed (Table 5). The high-specificity SCAR marker could detect the 360-bp amplification bands only in ‘Daboju’ samples, but not in the other chrysanthemum cultivar samples tested. After preliminary screening, the optimum annealing temperature for the SCAR-PCR reaction was determined to be 62 °C. SCAR-PCR was performed in a 20 μL reaction mixture containing 10 μL of 2×*EasyTaq* PCR SuperMix (Beijing TransGen Biotech Co., Ltd., Beijing, China), 1 μL of genomic DNA template (50 ng), 1 μL of forward primer (10 μM), 1 μL of reverse primer (10 μM), and 7 μL of ddH_2_O. PCR reaction was performed under the following conditions: 94 °C for 5 min, 35 cycles of 94 °C for 1 min, 62 °C for 50 s, and 72 °C for 1.5 min, followed by 72 °C extension for 10 min. The conditions of agarose gel (1.5%, *w/v*) electrophoresis were the same as described above.

### 4.8. Sensitivity and Application of the SCAR Marker

**Figure 8:**
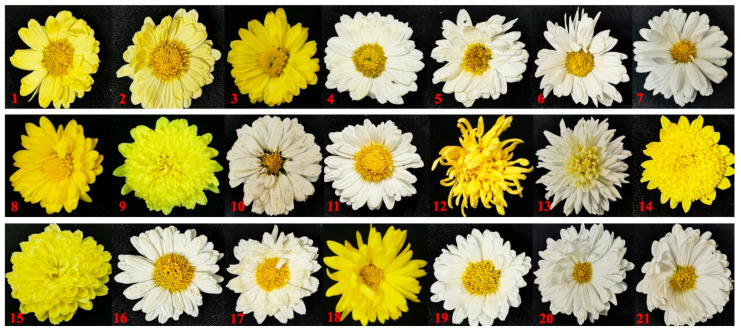
The flower morphology of 21 *C. morifolium* cultivars (1–21). Details of 21 *C. morifolium* cultivars are provided in Table 4.

**Figure 9:**
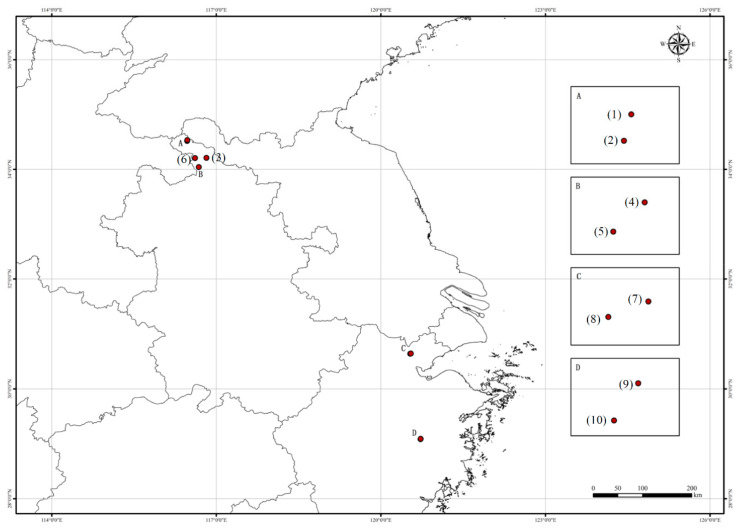
Sampling locations of 10 *C. morifolium* ‘Daboju’ populations. Numbers 1–10 are the same as those in Table 5.

The sensitivity of the SCAR marker (DBJF/DBJR) was tested with a dilution series of purified ‘Daboju’ genomic DNA: 50 ng, 40 ng, 30 ng, 20 ng, 10 ng, 100 pg, and 10 pg in 20 μL of PCR reaction mixture. To further test the applicability of the SCAR marker primer pairs (DBJF/DBJR) in practice, genomic DNA of ‘Xiaoyangju,’ ‘Zaoxiaoyangju,’ ‘Dayangju,’ ‘Machengju,’ and ‘Hongxinju,’ the flowers of which are similar to those of ‘Daboju’ (Figure 8), were utilized to simulate different adulterations of ‘Daboju’ (Table 4). The composition of the PCR reaction mixture, and conditions of the PCR amplification program and of agarose gel (1.5%, w/v) electrophoresis were the same as described above.

## 5. Conclusions

A ‘Daboju’-specific DNA fragment, SCoT36-385, was obtained, and a reliable and reproducible SCAR marker primer pair (DBJF/DBJR) was subsequently developed from this fragment for the identification of genuine samples of ‘Daboju’. The SCAR marker DBJF/DBJR could specifically identify genetic material of ‘Daboju’ and distinguish it from those of other *Chrysanthemum* cultivars. Thus, the development of these efficient SCAR markers for ‘Daboju’ will greatly contribute to the conservation and rational utilization of this important *Chrysanthemum* plant resource.

## Figures and Tables

**Table 1:** Sequences and polymorphism information of the 21 SCoT primers.

Primer Code	Primer Sequence (5’-3’)	Working Annealing Temperature (°C)	No. of Amplified Loci	No. of Polymorphic Loci	Polymorphic Loci (%)
SCoT1	CAACAATGGCTACCACCA	50	13	10	77.00
SCoT2	CAACAATGGCTACCACCC	52	12	12	100.00
SCoT3	CAACAATGGCTACCACCG	52	11	11	100.00
SCoT4	CAACAATGGCTACCACCT	50	7	6	85.70
SCoT5	CAACAATGGCTACCACGA	50	9	8	88.90
SCoT11	AAGCAATGGCTACCACCA	52	8	7	87.50
SCoT12	ACGACATGGCGACCAACG	56	7	5	71.40
SCoT16	ACCATGGCTACCACCGAC	54	9	8	88.90
SCoT19	ACCATGGCTACCACCGGC	57	10	8	80.00
SCoT20	ACCATGGCTACCACCGCG	58	10	9	90.00
SCoT21	ACGACATGGCGACCCACA	57	7	5	71.40
SCoT22	AACCATGGCTACCACCAC	52	6	4	66.70
SCoT23	CACCATGGCTACCACCAG	53	5	4	80.00
SCoT24	CACCATGGCTACCACCAT	52	9	9	100.00
SCoT26	ACCATGGCTACCACCGTC	54	10	9	90.00
SCoT30	CCATGGCTACCACCGGCG	60	10	10	100.00
SCoT32	CCATGGCTACCACCGCAC	56	10	9	90.00
SCoT33	CCATGGCTACCACCGCAG	56	7	6	85.70
SCoT34	ACCATGGCTACCACCGCA	56	7	5	71.40
SCoT35	CATGGCTACCACCGGCCC	58	12	12	100.00
SCoT36	GCAACAATGGCTACCACC	52	8	6	75.00
Average	-	-	8.90	7.76	85.70
Total	-	-	187	163	-

**Table 2:** Characteristics of the cultivar-specific SCAR primer pair derived from the cloned SCoT36-derived amplicon of *C. morifolium* ‘Daboju’.

SCAR Primer	SCAR Primer Sequence (5’-3’)	Length	GC Content (%)	Working Annealing Temperature (°C)	Amplicon Length (bp)
DBJF	ACCACCCAGGGAGGATTG	18	61.11	62	360
DBJR	CCACCTTGTAAGGCATGT	18	50.00		

**Table 3:** Simulated adulteration samples used for SCAR marker DBJF/DBJR application.

Num.		Different Incorporation Ratios of Chrysanthemum Varieties (%)					The Total Proportion of Non-‘Daboju’ Chrysanthemum Variety Adulteration (%)
	‘Daboju’	‘Xiaoyangju’	‘Zaoxiaoyangju’	‘Dayangju’	‘Machengju’	‘Hongxinju’	
1	100						0
2	50	50					50
3	33.3	33.3	33.3				67
4	25	25	25	25			75
5	20	20	20	20	20		80
6	0	20	20	20	20	20	100

**Table 4:** Details of *Chrysanthemum morifolium* samples used in the study.

Sample No.	Cultivar Name	Code	Material Types	Voucher Number	Location
1	*C. morifolium* ‘Jinju 1’	Jinju 1	Hangju	CmTx0001	Tongxiang, Zhejiang Province, China
2	*C. morifolium* ‘Jinju 2’	Jinju 2	Hangju	CmTx0002	Tongxiang, Zhejiang Province, China
3	*C. morifolium* ‘Jinju 3’	Jinju 3	Hangju	CmTx0003	Tongxiang, Zhejiang Province, China
4	*C. morifolium* ‘Xiaoyangju’	Xiaoyangju	Hangju	CmTx0004	Tongxiang, Zhejiang Province, China
5	*C. morifolium* ‘Zaoxiaoyangju’	Zaoxiaoyangju	Hangju	CmTx0005	Tongxiang, Zhejiang Province, China
6	*C. morifolium* ‘Dayangju’	Dayangju	Hangju	CmTx0006	Tongxiang, Zhejiang Province, China
7	*C. morifolium* ‘Yizhongdabaiju’	Yizhongdabaiju	Hangju	CmTx0007	Tongxiang, Zhejiang Province, China
8	*C. morifolium* ‘Xiaohuangju’	Xiaohuangju	Hangju	CmTx0008	Tongxiang, Zhejiang Province, China
9	*C. morifolium* ‘Huangyaoju’	Huangyaoju	Gongju	CmTx0009	Tongxiang, Zhejiang Province, China
10	*C. morifolium* ‘Jiuyueju’	Jiuyueju	Hangju	CmTx0010	Tongxiang, Zhejiang Province, China
11	*C. morifolium* ‘zhaohua 1’	Zhaohua 1	Hangju	CmTx0011	Tongxiang, Zhejiang Province, China
12	*C. morifolium* ‘Jinsihuangju’	Jinsihuangju	Jinsihuangju	CmXs0001	Xiushui, Jiangxi Province, China
13	*C. morifolium* ‘Daboju’	Daboju	Boju	CmBz0001	Bozhou, An’hui Province, China
14	*C. morifolium* ‘Zaogongju’	Zaogongju	Gongju	CmSx0001	Shexian, An’hui Province, China
15	*C. morifolium* ‘Wuyuanhuangju’	Wuyuanhuangju	Wuyuanhuangju	CmWy0001	Wuyuan, Jiangxi Province, China
16	*C. morifolium* ‘Xiaobaiju’	Xiaobaiju	Hangju	CmSy0001	Sheyang, Jiangsu Province, China
17	*C. morifolium* ‘Machengju’	Machengju	Machengju	CmMc0001	Macheng, Hubei Province, China
18	*C. morifolium* ‘Dahuangju’	Dahuangju	Hangju	CmSy0002	Sheyang, Jiangsu Province, China
19	*C. morifolium* ‘Hongxinju’	Hongxinju	Hangju	CmSy0003	Sheyang, Jiangsu Province, China
20	*C. morifolium* ‘Changbanju’	Changbanju	Hangju	CmSy0004	Sheyang, Jiangsu Province, China
21	*C. morifolium* ‘Dabaiju’	Dabaiju	Hangju	CmSy0005	Sheyang, Jiangsu Province, China

**Table 5:** List of *C. morifolium* ‘Daboju’ populations used for SCAR marker validation.

Number	Individual Numbers	Voucher Number	Location
1	10	CmBz0015	Wuma, Bozhou, An’hui Province, China
2	12	CmBz0016	Wuma, Bozhou, An’hui Province, China
3	11	CmBz0036	Shatu, Bozhou, An’hui Province, China
4	8	CmBz0052	Shuanggou, Bozhou, An’hui Province, China
5	13	CmBz0061	Shuanggou, Bozhou, An’hui Province, China
6	8	CmBz0074	Zhaoqiao, Bozhou, An’hui Province, China
7	10	CmTx0025	Tongxiang, Zhejiang Province, China
8	9	CmTx0032	Tongxiang, Zhejiang Province, China
9	10	CmPa0001	Pan’an, Zhejiang Province, China
10	12	CmPa0013	Pan’an, Zhejiang Province, China

## Data Availability

All data are included in the manuscript, and the specific DNA fragment, namely SCoT36-385, has been deposited in GenBank (GenBank accession number: OL456174).
